# Ultralow Lattice Thermal Conductivity of Zintl‐Phase CaAgSb Induced by Interface and Superlattice Scattering

**DOI:** 10.1002/smsc.202400147

**Published:** 2024-09-17

**Authors:** Wenhua Xue, Jie Chen, Honghao Yao, Jun Mao, Chen Chen, Yumei Wang, Qian Zhang

**Affiliations:** ^1^ School of Materials Science and Engineering, and Institute of Materials Genome & Big Data Harbin Institute of Technology Shenzhen 518055 China; ^2^ Institute of Physics Chinese Academy of Sciences Beijing 100190 China; ^3^ School of Physical Sciences Great Bay University Dongguan 523000 China; ^4^ Beijing Branch of Songshan Lake Materials Laboratory Beijing 100190 China

**Keywords:** CaAgSb, interfaces, superlattice structures, ultralow lattice thermal conductivities, Zintl phases

## Abstract

Zintl phases attract extensive attention due to the characteristic of “electron‐crystal, phonon glass”. In this work, an ultralow lattice thermal conductivity ≈0.59 W m^−1^ K^−1^ at 300 K and ≈0.3 W m^−1^ K^−1^ at 623 K is obtained in CaAgSb Zintl phase, which is much lower than that of other well‐known Zintl compounds. The origin of this ultralow lattice thermal conductivity is explored through first‐principles calculations and *C*_*s*_‐corrected scanning transmission electron microscopy. Theoretical phonon calculations provide evidence for complex phonon characteristics such as avoided‐crossing effect and low‐frequency flat band that favor the low lattice thermal conductivity. Moreover, subsequent microstructure results reveal abundant structural defects created in the CaAgSb sample, including superlattice structure and interface structure, which further contribute to the ultralow lattice thermal conductivity.

## Graphical abstract

The origin of ultralow *κ*_L_ in CaAgSb prepared by ball milling is revealed. The avoided‐crossing phenomenon and low‐frequency flat phonon bands are in favor of the ultralow *κ*_L_. Moreover, the intrinsic segregation of Ca and interface structure induced by ball milling can enhance the phonon scattering, further contributing to the ultralow *κ*_L_ within the whole temperature range.

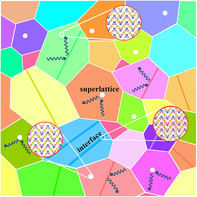

## 1 Introduction

Thermoelectric materials can realize the direct conversion of waste heat into electricity.^[^ ^1^, ^2^ ^]^ The performance of the thermoelectric material is governed by the dimensionless figure of merit *zT*, defined as *zT* = *S* ^2^ *σT*/*κ*, where *S* is the Seebeck coefficient, *σ* the electrical conductivity, *T* the absolute temperature, and *κ* the thermal conductivity that includes the electronic (*κ*_e_) and lattice (*κ*_L_) contributions: *κ* = *κ*_e_ + *κ*_L_.^[^ ^3^, ^4^, ^5^ ^]^ It is quite difficult to enhance the *zT* value of a thermoelectric material since *S* and *σ* are strongly coupled via the carrier concentration. The *κ*_L_ is the only parameter that can be regulated independently. Therefore, it is of significance to search for thermoelectric materials with intrinsic low *κ*_L_, arising from complex structure, large atomic weight, strong anharmonicity, or liquid‐like (atomic rattling) behavior, such as SnSe, Ag_8_SnSe_6_, Yb_14_MnSb_11_, Cu_2_S, etc. In addition, structural defects have been proven to effectively enhance phonon scattering and decrease the *κ*_L_, such as point defects,^[^ ^6^, ^7^ ^]^ grain boundaries,^[^ ^8^, ^9^, ^10^ ^]^ dislocations,^[^ ^11^, ^12^ ^]^ and nanostructures.^[^ ^13^, ^14^ ^]^ For example, the *κ*_L_ of PbTe can reach ≈0.5 W m^−1 ^K^−1^ at 850 K by incorporating SrTe nanocrystals.^[^ ^14^ ^]^ The nanotwins in InSb can scatter the phonons and reduce the *κ*_L_ from 13.6 to 8.2 W m^−1 ^K^−1^ at 300 K.^[^ ^15^ ^]^ The dislocations and nanodomains induced by actively controlling the Ge vacancies in GeTe‐based compounds favor the phonon scattering and eventually lead to the low *κ*_L_ of ≈0.48 W m^−1 ^K^−1^ at 648 K.^[^ ^16^ ^]^

Zintl phases have relatively low *κ*_L_ due to the complex crystal structure, inhomogeneous bonding, as well as the multidimensional defects. For example, the 5‐2‐6‐type Ca_5_Al_2_Sb_6_ compound possesses a complex structure and subsequently an extremely low lattice thermal conductivity ≈0.6 W m^−1 ^K^−1^ at 850 K.^[^ ^17^ ^]^ The 9‐4‐9‐type Yb_9_Mn_4.2_Sb_9_ compound has an ultralow *κ*_L_ of 0.45 W m^−1^ K^−1^ at room temperature due to both its large unit cell (44 atoms) and the large degree of disorder on the interstitial Mn site.^[^ ^18^ ^]^ The glass‐like *κ*_L_ ≈0.41 W m^−1 ^K^−1^ at 300 K is obtained in 2‐1‐2‐type Eu_2_ZnSb_2_ with a high‐symmetry structure but 50% intrinsic Zn vacancies and widely distributed plane defects.^[^ ^19^ ^]^ The partially occupied 1‐1‐1‐type CaZn_0.4_Ag_0.2_Sb Zintl phase with the LiGaGe structure also exhibits an ultralow *κ*_L_ (≈0.40 W m^−1 ^K^−1^) in the whole measured temperature range due to the widely distributed complex defects.^[^ ^20^, ^21^ ^]^

Herein, we prepared the 1‐1‐1‐type CaAgSb sample by high‐energy ball milling and hot pressing, achieving an ultralow *κ*_L_ ≈0.59 W m^−1 ^K^−1^ at 300 K and ≈0.3 W m^−1^ K^−1^ at 623 K, significantly lower than previously reported values. However, a detailed explanation for the ultralow thermal conductivity in the CaAgSb crystal has not been provided so far. To investigate the origin of this phenomenon, we conducted first‐principles calculations and *C*_*s*_‐corrected scanning transmission electron microscopy (STEM) analysis. Our findings reveal strong phonon interactions in CaAgSb, which contribute to its low thermal conductivity. Furthermore, microstructural analysis reveals abundant structural defects in the CaAgSb sample, including superlattices and interfaces, which further reduce the *κ*_L_. This study highlights the significance of microstructural defects in determining the low lattice thermal conductivity of Zintl‐phase compounds.

## 2 Results and Discussion

**Figure 1:**
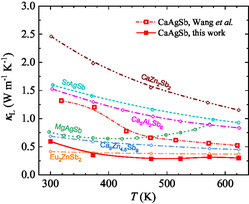
Temperature‐dependent *κ*_L_ for CaAgSb Zintl phase synthesized by ball milling and hot pressing. Temperature‐dependent *κ*_L_ for CaAgSb prepared by melting;^[^ ^20^, ^27^ ^]^ SrAgSb,^[^ ^24^ ^]^ MgAgSb,^[^ ^26^ ^]^ CaZn_2_Sb_2_,^[^ ^22^ ^]^ Ca_5_Al_2_Sb_6_,^[^ ^17^ ^]^ Eu_2_ZnSb_2_,^[^ ^19^ ^]^ and Ca_9_Zn_4.5_Sb_9_^[^ ^7^ ^]^ Zintl compounds are included for comparison.

The lattice thermal conductivity *κ*_L_ as a function of temperature for CaAgSb is presented in **Figure** **1** with other well‐known Zintl compounds for comparison. The 1‐2‐2‐type CaZn_2_Sb_2_ processes the relatively low *κ*_L_ of ≈2.46 W m^−1^ K^−1^ at 300 K.^[^ ^22^ ^]^ The ZrBeSi‐type SrAgSb compound owns an abnormally low *κ*_L_ of ≈1.78 W m^−1^ K^−1^ at 300 K due to the soft and anharmonic lowest‐lying optical (LLO) mode.^[^ ^23^, ^24^ ^]^ Similar to SrAgSb, the low *κ*_L_ in Ca_5_Al_2_Sb_6_ appears to arise from the low group velocity of the optical modes and Umklapp scattering of the acoustic modes.^[^ ^17^ ^]^ Both global and local weak chemical bonds in *α*‐MgAgSb result in “rattling‐like” thermal damping to reduce lattice thermal conductivity to ≈0.76 W m^−1^ K^−1^ at 300 K.^[^ ^25^, ^26^ ^]^ A lower *κ*_L_ of ≈0.68 W m^−1^ K^−1^ at 300 K is observed in 9‐4‐9‐type Ca_9_Zn_4.5_Sb_9_ due to its complex crystal structure.^[^ ^7^ ^]^ Meanwhile, 2‐1‐2‐type Eu_2_ZnSb_2_ shows an averaged ultralower *κ*_L_ ≈0.38 W m^−1^ K^−1^ within the whole measured temperature range.^[^ ^19^ ^]^ It is worth noting that the *κ*_L_ of CaAgSb prepared in this work keeps a decreasing trend with rising temperature from ≈0.59 W m^−1^ K^−1^ at 300 K to ≈0.3 W m^−1^ K^−1^ at 623 K, much lower than that of the CaAgSb prepared by melting in the whole measured temperature range.^[^ ^20^, ^27^ ^]^ Such low thermal conductivity in CaAgSb implies an interesting phonon‐scattering mechanism that needs to be investigated.

**Figure 2:**
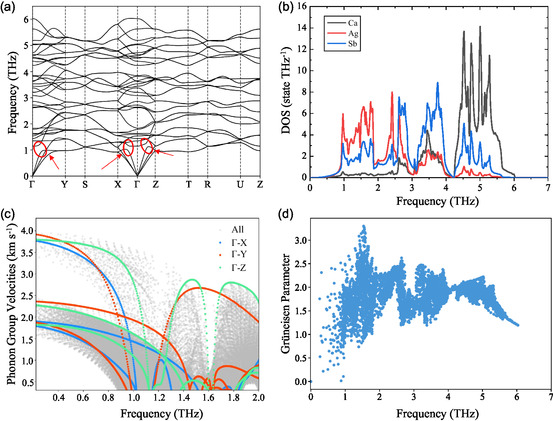
a) Phonon dispersion, b) density of states, c) group velocity, and d) Grüneisen parameters of CaAgSb. The direction along Γ—X, Γ—Y, and Γ—Z directions are colored with blue, red, and green, respectively.

To elucidate possible origin of the low *κ*_L_ in CaAgSb, we conducted first‐principles density‐functional theory (DFT) calculations on the phonon dispersions (**Figure** **2**a). We find several flat phonon branches in the range of 1–2 THz in the Brillouin zone. Another salient characteristic involves the phenomenon of avoided‐crossing interaction between the optical and acoustic branches, which is evidenced by well‐defined energy gaps separating the two spectral bands indicated by the red rings and arrows. Those avoided‐crossing zones originate from the Γ point along the X, Y, and Z points occurring around 1 THz. The large difference in atomic interaction or atomic masses can result in a significant anti‐crossings phenomenon based on a linear chain model.^[^ ^28^, ^29^ ^]^ In CaAgSb materials, calculations show that Ag atoms have a large mean square displacement (Figure S1, Supporting Information), indicating a shallow energy surface and reduced force constants. Additionally, crystal orbital Hamilton population calculations^[^ ^30^ ^]^ (Figure S2, Supporting Information) reveal that Ag—Sb bonds exhibit occupied antibonding states near the valence band maximum, which effectively weakens the bonding. Weak bonding around Ag atoms could induce this avoided‐crossing phenomenon. The phonon density of states shows that Ag and Sb atoms account for the main contribution at the low‐frequency zone, while Ca atoms dominate at the high‐frequency zone (Figure 2b).

The calculated longitudinal *v*_L_ and transverse *v*_T_ sound velocities are 3.8 and 2.0 km s^−1^ based on the phonon dispersion around Γ point, respectively. We calculate the average of 5% of the reciprocal lattice vectors along the Γ—X, Γ—Y, and Γ—Z directions starting from the Γ point. Each branch velocity is averaged by all directions and detailed values are listed in Table S1, Supporting Information. The total average sound velocity is 2.26 km s^−1^ at room temperature. The sound velocity of CaAgSb is higher than that of many well‐known thermoelectric materials, such as PbTe^[^ ^31^, ^32^ ^]^ (1.78 km s^−1^), GeTe^[^ ^33^ ^]^ (1.94 km s^−1^), and Bi_2_Te_3_^[^ ^34^, ^35^ ^]^ (1.62 km s^−1^). Meanwhile, CaAgSb possesses a much lower thermal conductivity (≈0.4 W m^−1^ K^−1^, in this work) compared with PbTe^[^ ^31^, ^32^ ^]^ (2.0 W m^−1^ m^−1^), GeTe^[^ ^33^ ^]^ (2.8 W m^−1^ m^−1^), and Bi_2_Te_3_^[^ ^34^, ^35^ ^]^ (1.3 W m^−1^ m^−1^). The macroscopic sound velocities mean the relative strong bonding in the main structure. However, the large phonon gap raised by the avoided‐crossing effect breaks the extensions of the longitudinal acoustic and LLO phonon branches.^[^ ^36^, ^37^ ^]^ The calculated phonon group velocity is shown in Figure 2c. The colored group velocities contributed by phonon modes along Γ—X, Γ—Y, and Γ—Z directions show the trend of large reduction of group velocity around 1 THz, contributing to the low *κ*_L_. This can explain moderate macroscopic sound speed system can also has a low *κ*_L_.

Grüneisen parameters were also calculated and they increase until 1.5 THz as shown in Figure 2d. Such anharmonic interactions could also reduce thermal conductivity through reductions in phonon lifetimes via phonon–phonon anharmonic scattering, as shown in Figure S3, Supporting Information. The increased Grüneisen could reduce the lattice thermal conductivity of CaAgSb. In addition, some research works have found the low‐frequency flat phonon bands can also significantly increase phonon–phonon scattering rates,^[^ ^38^ ^]^ detailly in providing more scattering channels^[^ ^39^, ^40^, ^41^ ^]^ just based on the shape of phonon bands.

**Figure 3:**
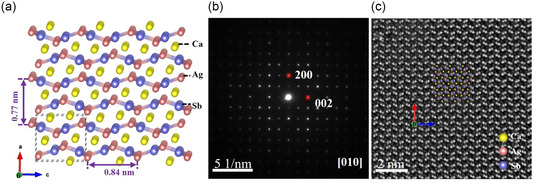
a) Crystal structure of CaAgSb viewed along the *b* axis, the Ca, Ag, and Sb atoms are yellow, red,. and blue spheres, respectively. b) [010] SAED pattern and c) [010] HAADF–STEM image of CaAgSb prepared by ball milling. Crystal structures is visualized by VESTA.^[^ ^51^ ^]^

Apart from the theoretical calculation, the microstructural investigation was performed to further understand the lower *κ*_L_ of CaAgSb prepared by ball milling. **Figure** **3**a shows the crystal structure of CaAgSb, in which the Ca, Ag, and Sb atoms are yellow, red, and blue spheres, respectively. The selected area electron diffraction (SAED) pattern shown in Figure 3b is indexed by [010] axis of CaAgSb. In the high‐angle annular dark field (HAADF)–STEM image, the atomic columns of Ca, Ag, and Sb are separately distinguishable (Figure 3c), where the brightest dots represent the Sb atomic columns, the moderate ones are the Ag atomic columns, and the weak ones are the Ca atomic columns due to the HAADF–STEM image contrast proportional to *Z* ^1.7^(*Z* is the atomic number), matching well with the [010] structural projection of CaAgSb.

**Figure 4:**
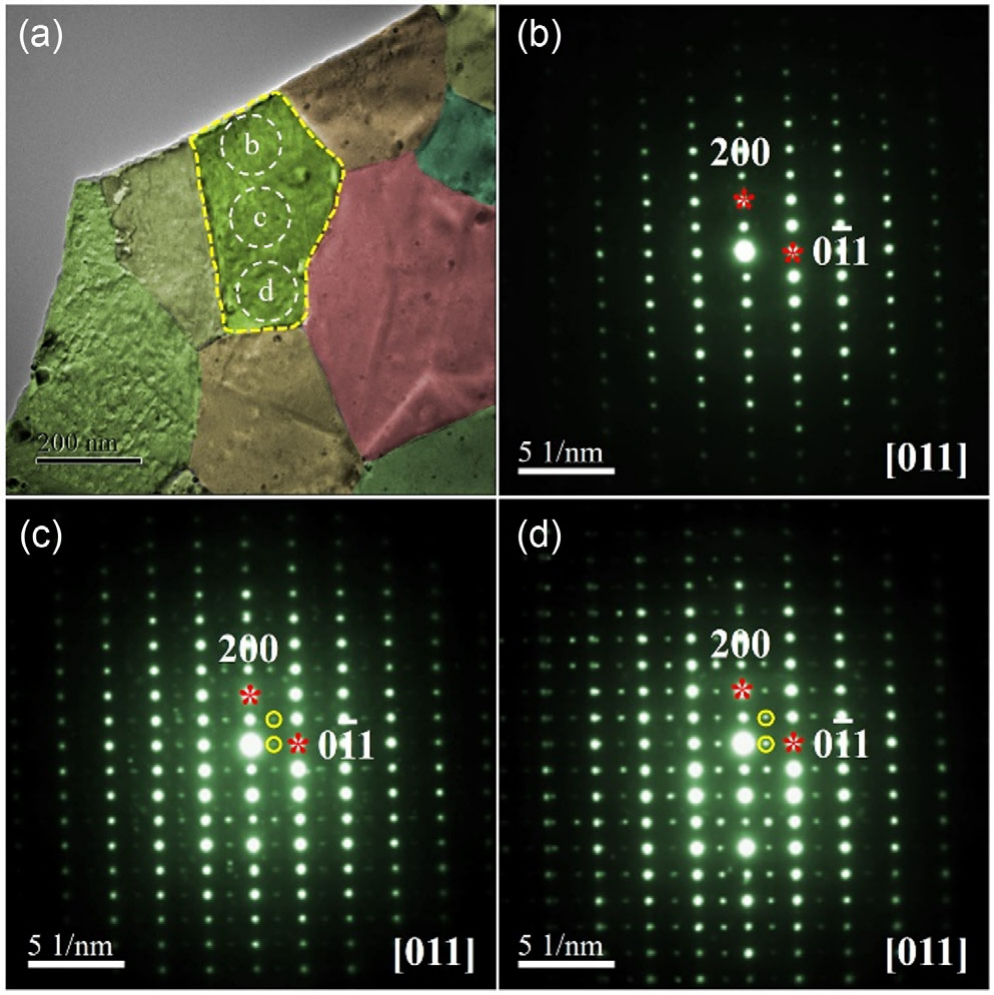
a) The typical low‐magnification TEM images of the CaAgSb sample prepared by ball milling, b–d) [011] SAED patterns taken from regions b, c, and d marked by white circles in (a), respectively. The main spots were indicated by red stars, and the modulation spots were indicated by yellow circles.

The typical low‐magnification transmission electron microscopy (TEM) image of CaAgSb sample is shown in **Figure** **4**a. The CaAgSb compound is well crystallized with the grain size of 200–500 nm. Figure 4b–d presents the corresponding SAED patterns of the regions b, c, and d indicated by white circles in Figure 4a, respectively. The SAED patterns corresponding to regions b, c, and d show evident difference, in which the SAED pattern in Figure 4b can be indexed by the [011] axis of the CaAgSb with the orthorhombic structure. However, the SAED patterns (Figure 4c,d) notably demonstrate two sets of electron diffraction spots. One set is the main spots with the stronger contrast (indicated by stars), which can be indexed by the [011] axis of the CaAgSb sample. And the other set is the modulation spots with weaker contrast indicated by circles. The origin of the modulation spots will be clarified later from the high‐resolution images. Interestingly, the modulation spots in Figure 4d are obviously stronger than those in Figure 4c with the modulation spots gradually strengthening from Figure 4b–d.

**Figure 5:**
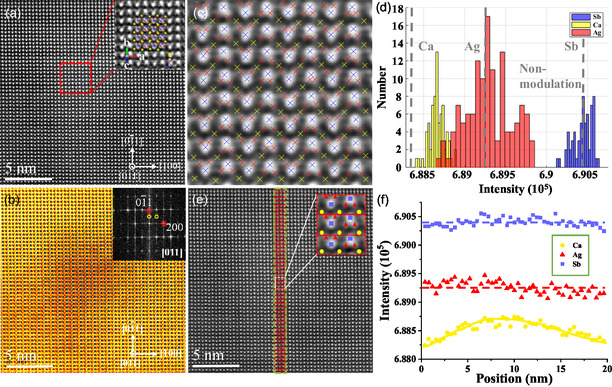
a) HAADF–STEM image of the circular region c in Figure 4a with the magnification of the region marked by the red rectangular inserted. b) ABF‐STEM image corresponding to (a) with insert FFT. c) The magnification of rectangular region in (a) with blue, red, and yellow × representing Sb, Ag, and Ca atom. d) Intensity statistics of Sb, Ag, and Ca atoms in (c). The three gray‐dashed lines from left to right representing the average atomic intensities of Ca, Ag, and Sb in the non‐modulated region, respectively. e) The green‐dashed rectangular across the modulation and non‐modulation region in (a), with each red box representing a unit. f) Average intensities of Ca, Ag, and Sb of units from top to bottom in (e).

The HAADF–STEM image and annular bright field (ABF)–STEM image taken from region c in Figure 4a are shown in **Figure** **5**a,b, respectively. The contrast of central region is more prominent compared with that around. The fast Fourier ransform (FFT) for the red rectangle region in Figure 5a is inserted in Figure 5b. Apart from the main spots indexed by the [011] axis, there are another set of weak spots, consistent with those shown in Figure 4c,d. The weaker modulation spots (indicated by circles) in the $\text{[0}\bar{1}\text{1]}$ direction is located at half of the main spots (indicated by stars). Generally, the origin of the modulation spots arises from the doubling of the periodicity of crystal structure. However, the periodic atomic displacement has not been observed in the HAADF–STEM image of CaAgSb. Therefore, it is very likely that the periodic intensity changes of Ca, Ag, or Sb results in double modulation in the $\text{[0}\bar{1}\text{1]}$ direction.

To reveal the modulation spots, a statistical investigation on the intensity of all atoms in the red rectangular region (modulation spots appeared) in Figure 5a was performed using the CalAtom software.^[^ ^42^ ^]^ Figure 5c is a magnified image of the red rectangular region, where blue, red, and yellow × represent Sb, Ag, and Ca atom with the intensity counted as shown in Figure 5d, in which the three gray‐dashed lines from left to right represent the average atomic intensities of Ca, Ag, and Sb in the non‐modulated region, respectively. The statistical intensities show no significant difference for Ag and Sb between the modulated and non‐modulated region, while the intensities of Ca in the modulated region exceed that in the non‐modulated region. Therefore, the segregation of Ca gives rise to the appearance of modulation spots.

To clarify the segregation of Ca, the intensities of Ca, Ag, and Sb across the modulation and non‐modulation region marked with green rectangular in Figure 5e were calculated. Each red box represents a unit (inserted in Figure 5e), in which the intensities of Ca, Ag, and Sb were averaged respectively and then counted from top to bottom, as presented in Figure 5f. The intensities of Sb and Ag hardly fluctuate, while the intensities of Ca deviates in the modulation region with strengthening first and then weakening from top to bottom. Therefore, the inhomogeneous segregation of Ca decreases gradually from the central to the periphery, which also clarifies the intensity differences of modulation spots in Figure 4. The superlattice structures caused by intrinsic segregation of Ca can act as effective scattering centers for the low‐frequency phonons similar to nanostructures, leading to the *κ*_L_ of CaAgSb at an ultralow level and providing a guarantee for the subsequent improvement of thermoelectric performance.

**Figure 6:**
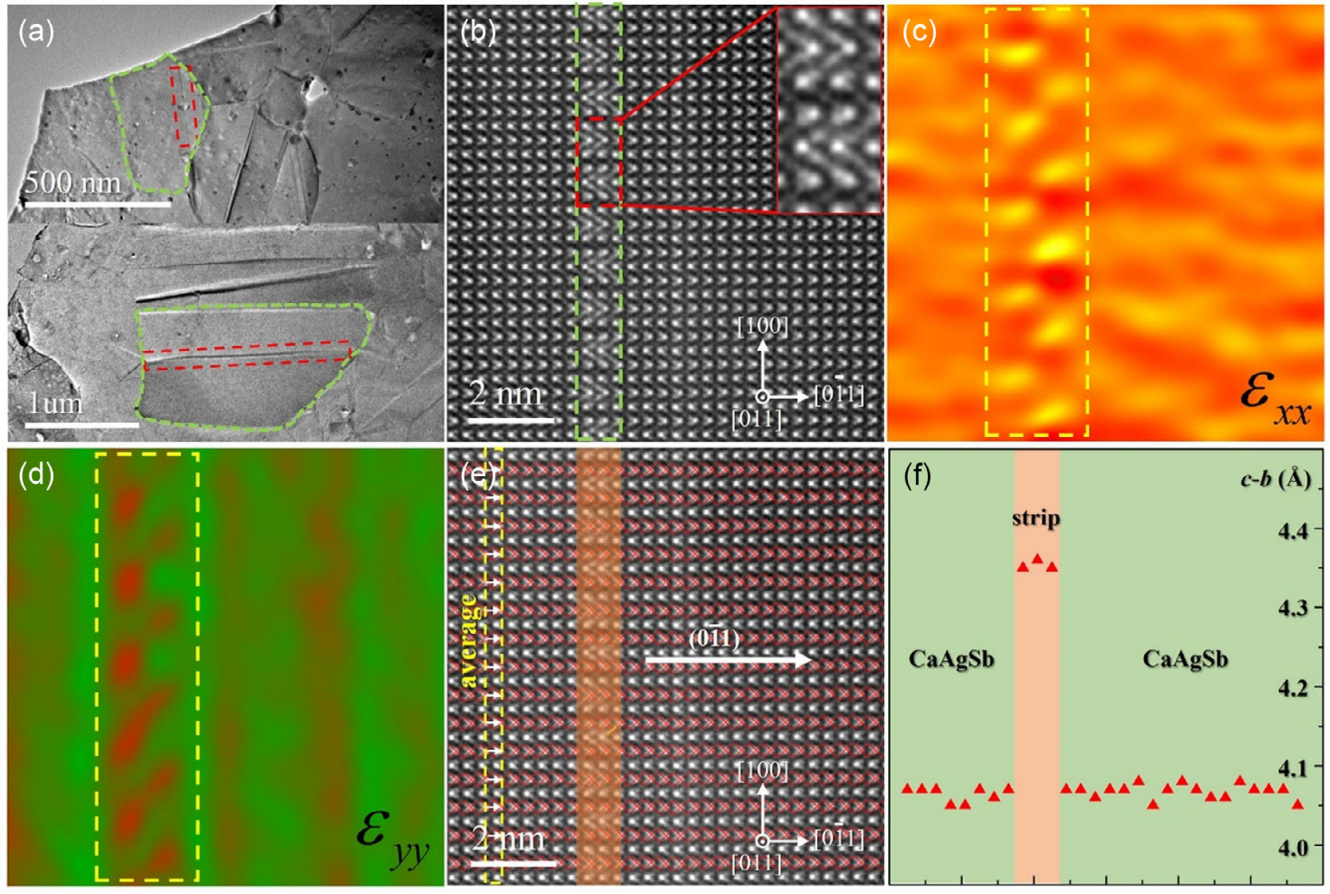
a) Low‐magnification TEM images of the CaAgSb sample, strip contrasts indicated by red rectangles. b) HAADF–STEM image with sharp strip contrast indicated by the green rectangle. c,d) GPA images of (b) along the in‐plane and out‐of‐plane directions, respectively. e) HAADF–STEM image with the yellow‐dashed rectangle representing a large unit. f) Averaged $\text{(0}\bar{1}\text{1)}$ lattice parameters of large units from left to right in (e).

In addition, numerous strip contrasts marked by red rectangles are clearly observed in different crystal grains of the CaAgSb sample prepared by ball milling (**Figure** **6**a). The HAADF–STEM image taken from the strip region in Figure 6a is shown in Figure 6b, in which the 3‐unit‐cell thick interface structure is marked by the green rectangular magnified in the upper right along the $\text{[0}\bar{1}\text{1]}$ direction. The “Geometric Phase Analysis” (GPA) plugin is used in the DigitalMicrograph software to study the strain field around the interface structure. Figure 6c,d shows the GPA of Figure 6b along the in‐plane and out‐of‐plane directions. The strong stress concentration indicates a large lattice distortion in the interface region. We measured the $\text{(0}\bar{1}\text{1)}$ lattice parameters of interface region in Figure 6b with CalAtom software. The region marked by yellow‐dashed rectangle in Figure 6e was regarded as a large unit, in which the measured $\text{(0}\bar{1}\text{1)}$ lattice parameters are averaged. The results of averaged $\text{(0}\bar{1}\text{1)}$ lattice parameters from left to right in Figure 6e are shown in Figure 6f. The lattice parameter for the parent phase (CaAgSb) is ≈4.07 Å, while that for the interface region (marked with red band) is ≈4.35 Å. Therefore, stretching lattice results in the interface structure with $\text{(0}\bar{1}\text{1)}$ lattice parameters exceeding parent phase (CaAgSb) 0.28 Å. The interface structure can effectively serve as phonon scattering source, which is beneficial to the lower *κ*_L_, and also proves that CaAgSb has great potentiality for subsequent thermoelectric property optimization.

## 3 Conclusion

In summary, we explored the origin of ultralow *κ*_L_ in CaAgSb prepared by ball milling. It reveals that the complex phonon band characteristics (avoided‐crossing effect and low‐frequency flat phonon bands) are in favor of the ultralow *κ*_L_. Moreover, the rich microstructures, including the intrinsic segregation of Ca and interface structure induced by ball milling, can enhance the phonon scattering, further contributing to the ultralow *κ*_L_ within the whole temperature range. Our results advance the understanding of low *κ*_L_ in Zintl‐phase CaAgSb and demonstrate great potentiality for the subsequent property optimization.

## 4 Experimental Section

Calcium (Ca, 99.9%, shots, Alfa Aesar), silver (Ag, 99.9%, shots, Alfa Aesar), and antimony (Sb, 99.999%, shots, ZhongNuo Advanced Material) were weighed according to the stoichiometry of CaAgSb. For the ball‐milling experiment, the raw elements were loaded into a stainless‐steel jar in an argon‐filled glove box, and then ball‐milled continuously for 10 h using a high‐energy ball mill (SPEX 8000M). The obtained powder was sintered into a dense disk at 973 K for 3 min with spark plasma sintering under an axial pressure of 60 MPa.

The crystal structure was analyzed by X‐Ray diffraction (Rigaku D/max 2500 PC, with Cu K*α* radiation *λ* = 1.5418 Å). The obtained sample was single phase without any impurity phase as shown in Figure S4, Supporting Information. Specimens used for TEM observation were prepared by traditional mechanical polishing, dimpling, and then ion milling with liquid‐nitrogen stage. TEM and HAADF–STEM investigations were carried out under 200 kV by using JEM‐ARM 200 F equipped with a cold field emission gun source and double‐*C*_s_ correctors respectively. The attainable spatial resolution of the microscope was 80 pm. HAADF images were acquired at acceptance angles of 70–150 mrad. All the HAADF images presented in this work were Fourier filtered to reduce the impact of irregular noise.

The thermal conductivity (*κ*) was calculated from *κ* = *DαC*_p_, where *D* is the volumetric density (5.95 g cm^−3^) determined by the Archimedes method with the relative density of 98%, *α* is the thermal diffusivity measured on a laser flash apparatus (Netzsch LFA457), and *C*_p_ is the specific heat estimated by Dulong–Petit law. The Seebeck coefficient (*S*) and electrical conductivity (*σ*) were measured at the same time by using the CTA‐3 (Cryoall, China). The *κ*_L_ was obtained by subtracting the carrier thermal conductivity *κ*_e_ from the total thermal conductivity *κ*. The *κ*_e_ was estimated using Wiedemann–Franz relationship (*κ*_e_ = *LσT*), where *L* is the Lorenz number calculated using the single parabolic band model. The temperature‐dependent electrical conductivity, Seebeck coefficient, thermal diffusivity, and total thermal conductivity for CaAgSb synthesized by ball milling and hot pressing is presented in Figure S5, Supporting Information. The temperature‐dependent Lorenz number is shown in Figure S6, Supporting Information. The uncertainty for the electrical conductivity was 3%, the Seebeck coefficient 5%, the thermal diffusivity 4%, and the thermal conductivity 6%.

## 5 Calculation Method

The first‐principles calculation was employed within the framework of DFT with the help of VASP^[^ ^43^, ^44^, ^45^ ^]^ code. The generalized gradient approximation of Perdew, Burke, and Ernzerh^[^ ^46^ ^]^ was used as the exchange–correlation functionals. The plane‐wave basis projector‐augmented wave method was implemented under the energy cutoff of 500 eV. The second and third interatomic force constant was both calculated with the finite displacement method under the supercell size of 4 × 2 × 2. Reciprocal space was sampled in the γ‐center method with the size of 2 × 2 × 2. The phonon dispersion and postprocess were implemented in the Phonopy.^[^ ^47^ ^]^ The mode‐dependent Grüneisen parameters and phonon scattering rates were obtained from the third‐order force constant based on the literatures^[^ ^48^, ^49^ ^]^ by the ShengBTE^[^ ^50^ ^]^ code. The calculation parameters were considered with the eighth nearest neighbor, and the q‐mesh with 24 × 12 × 12 was used.

## Conflict of Interest

The authors declare no conflict of interest.

## Author Contributions

**Wenhua Xue**: Data curation (lead); Validation (lead); Visualization (lead); Writing—original draft (lead). **Jie Chen**: Data curation (lead); Formal analysis (lead); Visualization (lead); Writing—original draft (lead). **Honghao Yao**: Formal analysis (equal); Validation (equal); Writing—original draft (equal). **Jun Mao**: Funding acquisition (supporting); Writing—review and editing (supporting). **Chen Chen**: Formal analysis (equal); Resources (lead); Validation (lead); Writing—review and editing (equal). **Yumei Wang**: Data curation (lead); Funding acquisition (lead); Investigation (lead); Supervision (lead); Writing—review and editing (lead). **Qian Zhang**: Funding acquisition (lead); Resources (lead); Supervision (lead); Writing—review and editing (lead). Wenhua Xue, Jie Chen, and Honghao Yao have contributed equally to this work.

## Supporting information

Supplementary Material

## Data Availability

Research data are not shared.
