# Supplementary material for: Ultralow Lattice Thermal Conductivity of Zintl‐Phase CaAgSb Induced by Interface and Superlattice Scattering
Source: Small Sci. 2024 Sep 17;5(3):2400147. doi: 10.1002/smsc.202400147 (PMC12244999; doi:10.1002/smsc.202400147)
Supplement: Supplementary file 1 — Supplementary Material [file SMSC-5-2400147-s001.pdf]

Ultralow Lattice Thermal Conductivity of Zintl Phase CaAgSb Induced by Interface and Superlattice Scattering

Wenhua Xue,[1,2#] Jie Chen,[2#] Honghao Yao,[1#] Jun Mao,[1] Chen Chen,[3*] Yumei Wang,[2,4*] Qian Zhang[1*]

[1] School of Materials Science and Engineering, and Institute of Materials Genome & Big Data, Harbin Institute of Technology, Shenzhen 518055, China

[2] Institute of Physics, Chinese Academy of Sciences, Beijing 100190, China

[3]School of Physical Sciences, Great Bay University, Dongguan, 523000, China

[4]Beijing Branch of Songshan Lake Materials Laboratory, Beijing 100190, China

Table S1. The detailed and averaged velocities from derivative of phonon band near Γ-point. (km s^{-1})

	Γ-X	Γ-Y	Γ-Z	Average
v_{TA1}	1.84	1.91	1.82	2.02
v_{TA2}	1.91	2.37	2.3	
v_{LA}	3.79	3.89	3.8	3.83

$$v_{\mathrm{TA}} = \frac{v_{\mathrm{TA1(\Gamma-X)}} + v_{\mathrm{TA1(\Gamma-Y)}} + v_{\mathrm{TA1(\Gamma-Z)}} + v_{\mathrm{TA2(\Gamma-X)}} + v_{\mathrm{TA2(\Gamma-Y)}} + v_{\mathrm{TA2(\Gamma-Z)}}}{6} \quad (1)$$

$$v_{\mathrm{LA}} = \frac{v_{\mathrm{TL(\Gamma-X)}} + v_{\mathrm{TL(\Gamma-Y)}} + v_{\mathrm{TL(\Gamma-Z)}}}{3} \quad (2)$$

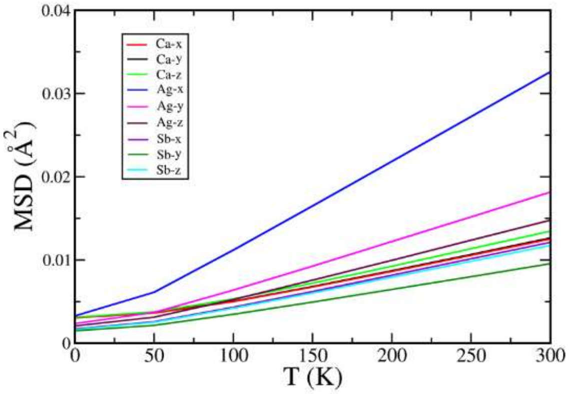

Fig. S1. Calculated mean square displacement (MSD) along the x, y, and z directionsfor CaAgSb.

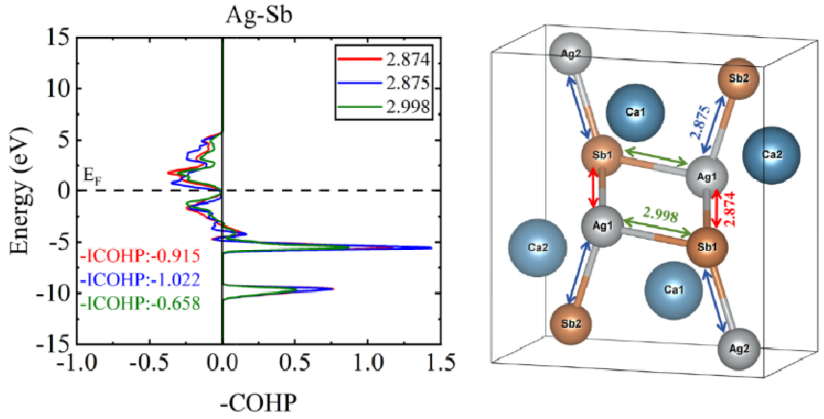

Fig. S2. Crystal orbital Hamilton population (COHP) for the nearest-neighbor Ag-Sb atomic pairs for CaAgSb.

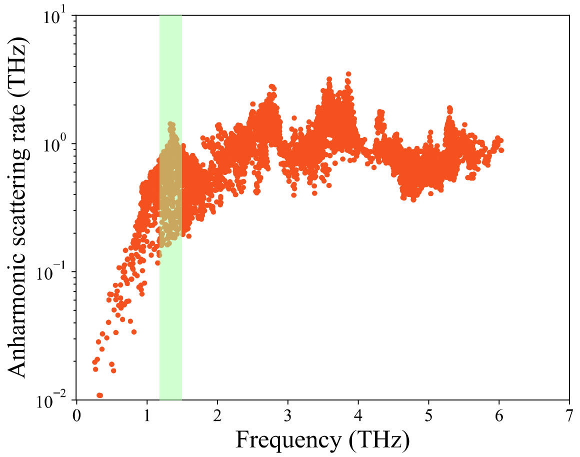

Fig. S3. Anharmonic scattering rate as a function of frequency for CaAgSb.

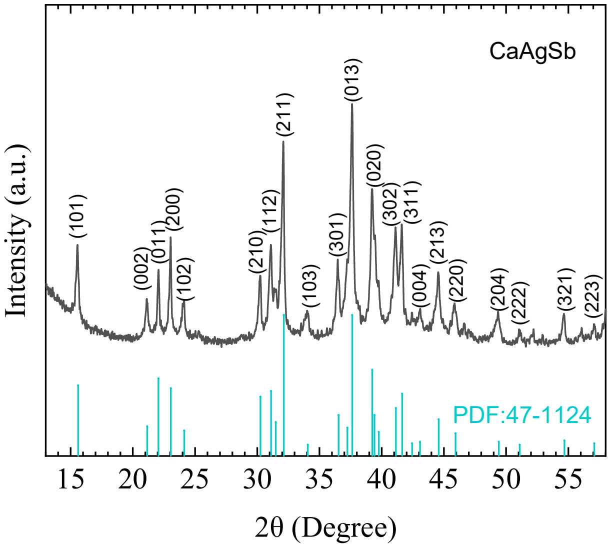

Figure S4. XRD pattern of CaAgSb.

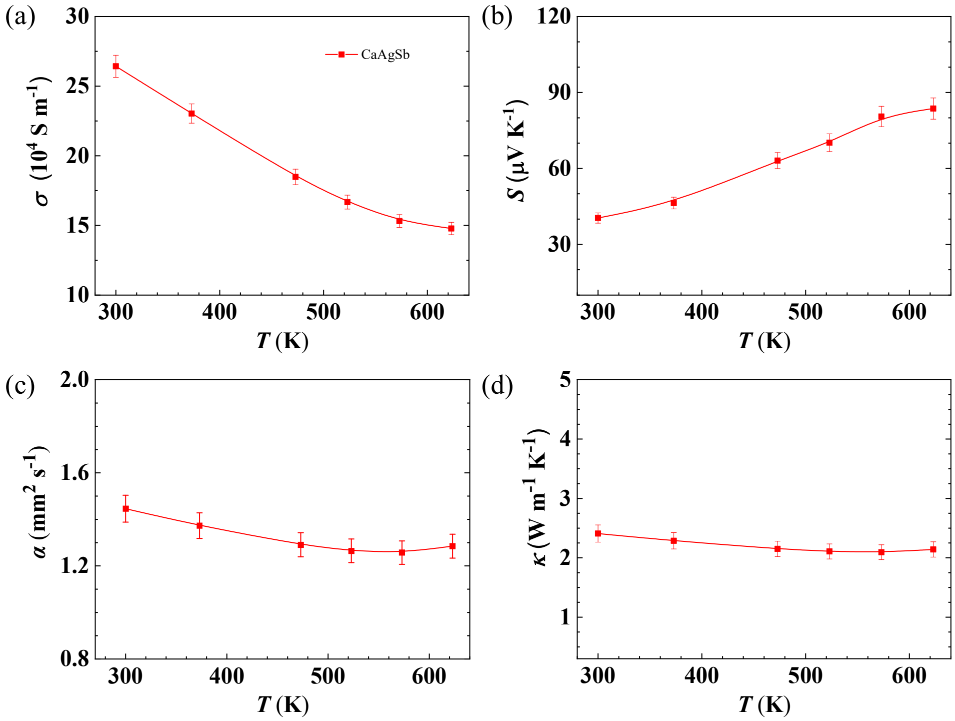

Figure S5. Temperature-dependent (a)Electrical conductivity, (b) Seebeck coefficient, (c)Thermal diffusivity, and (d) Total thermal conductivity for CaAgSb Zintl phase synthesized by ball milling and hot pressing.

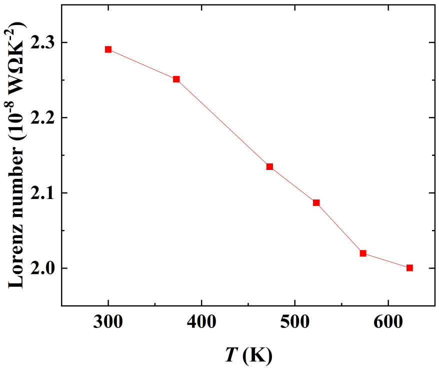

Figure S6. Temperature-dependent Lorenz number for CaAgSb Zintl phase synthesized by ball milling and hot pressing.

For the single parabolic model, the Lorenz number can be given as[1]:

$$L=\left(\frac{k_B}{e}\right)^2\left(\frac{(r+7/2)F_{r+5/2}(\eta)}{(r+3/2)F_{r+1/2}(\eta)}-\left[\frac{(r+5/2)F_{r+3/2}(\eta)}{(r+3/2)F_{r+1/2}(\eta)}\right]^2\right) \quad (3)$$

For the Lorenz number calculation, we should get reduced Fermi energy η firstly; the calculation of η can be derived from the measured Seebeck coefficients by using the following relationship:

$$S=\pm\frac{k_B}{e}\left(\frac{(r+5/2)F_{r+3/2}(\eta)}{(r+3/2)F_{r+1/2}(\eta)}-\eta\right) \quad (4)$$

where $F_n(\eta)$ is the n-th order Fermi integral,

$$F_n(\eta)=\int_0^\infty \frac{\chi^n}{1+e^{\chi-\eta}}d\chi \quad (5)$$

$$\eta=\frac{E_f}{k_B T} \quad (6)$$

In the above equations, k_B is the Boltzmann constant, e the electron charge and $E_f$the Fermi energy. Meanwhile, acoustic phonon scattering (r = -1/2) has been assumed as the main carrier scattering mechanism.

[1] Y.-L. Pei, J. He, J.-F. Li, F. Li, Q. Liu, W. Pan, C. Barreteau, D. Berardan, N. Dragoe and L.-D. Zhao, *NPG Asia Mater* **2013**,5,47.
